# Formylpeptide Receptors Promote the Migration and Differentiation of Rat Neural Stem Cells

**DOI:** 10.1038/srep25946

**Published:** 2016-05-13

**Authors:** Guan Wang, Liang Zhang, Xingxing Chen, Xin Xue, Qiaonan Guo, Mingyong Liu, Jianhua Zhao

**Affiliations:** 1 Department of Spine Surgery, Daping Hospital, Third Military Medical University, Chongqing 400042, China; 2 Department of Pathology, Xinqiao Hospital, Third Military Medical University, Chongqing 400037, China

## Abstract

Neural stem cells (NSCs) bear characteristics for proliferation, migration and differentiation into three main neural cell type(s): neurons, astrocytes and/or oligodendrocytes. Formylpeptide receptors (Fprs), belonging to the family of G protein-coupled chemoattractant receptors, have been detected on neurons in the central nervous system (CNS). Here, we report that Fpr1 and Fpr2 are expressed on NSCs as detected with immunohistochemistry, RT-PCR and WB assays. In addition, Fpr1 and Fpr2 promoted NSC migration through F-actin polymerization and skewed NSC differentiation to neurons. Our study demonstrates a unique role of Fpr1 and Fpr2 in NSCs and opens a novel window for cell replacement therapies for brain and spinal cord injury.

Neural stem cells (NSCs) bear the capacity of self-renewal and differentiation into three main neural cell type(s): neurons, astrocytes and oligodendrocytes^1^,^2^. NSCs are likely to proliferate at discrete niches (subventricular zone and subgranular zone), reroute toward lesions and integrate into damaged neuronal network following brain and spinal cord injury^3^,^4^,^5^. During pathological process, quiescent NSCs or NSCs in the special microenvironments receive signals from extracellular changes and migrate to their final positioning location and align to rebuild injured neurovascular network in response to chemokines and cytokines. In the last decade, accumulating evidence showed that immune system targets neurogenic niches and exerts a considerable effect on the proliferation, migration and differentiation of NSCs^6^,^7^,^8^,^9^,^10^,^11^.

Formylpeptide receptors (Fprs) belong to the family of G protein-coupled chemoattractant receptors. There are three family members, Fpr1 (FPR1 in human), Fpr2 (FPR2 in human) and Fpr3 (FPR3 in human)^12^,^13^,^14^,^15^. More recently, the expressions of these receptors have been demonstrated on other cell types, although most functional studies for Fprs were carried out using neutrophils and monocytes.

Increasing evidence indicate that Fprs, including Fpr1 and/or Fpr2, expressed in central nervous system (CNS) have the ability to interact with formyl-methyl-leucyl-phenylalanine (fMLF/fMLP)^16^,^17^, and these receptors have been detected in human brain, spinal cord, anterior horn cells and hypoglossal nucleus neurons^18^.

The interaction between chemotactic receptors and ligands (i.e. chemokines and cytokines) assists NSCs to recognize signals along the migration path towards their destination. For instance, CXCR4 expressed by NSCs interacts with the ligand stromal derived factor-1a (SDF-1a) to induce NSC migration following neural injury^19^. Whether NSCs express Fprs to mediate their migration and, proliferation, migration and differentiation remains unknown.

In the present study, we examined whether NSCs express Fpr1 and Fpr2 by immunocytochemistry, reverse transcription polymerase chain reaction (RT-PCR) and Western blotting (WB), also assessed their function on proliferation, migration and differentiation of NSCs by using fMLF (an Fpr1 agonist)^20^, tBOC (an Fpr1 antagonist)^21^, MMK-1 (an Fpr2 agonist)^22^ and WRW4 (an Fpr2 antagonist)^23^. The data may establish a new concept of the roles of Fprs in CNS.

## Results

### Both Fpr1 and Fpr2 are expressed by NSCs

**Figure 1:**
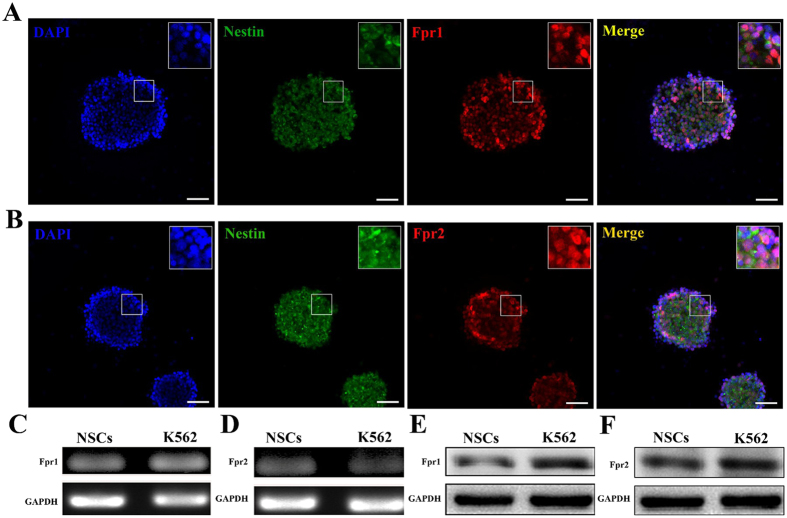
The expression of Fpr1 and Fpr2 in NSCs. (**A**) Immunocytochemistry for the expression of Fpr1 (red), co-labeled with Nestin (green) and DAPI (blue) (n = 3). (**B**) Immunocytochemistry for the expression of Fpr2 (red), co-labeled with Nestin (green) and DAPI (blue) (n = 3). (**C**) The expression of Fpr1 with K562cell line as positive control by RT-PCR assay (n = 3). (**D**) The expression of Fpr2 with K562cell line as positive control by RT-PCR assay (n = 3). (**E**) The expression of Fpr1 with K562cell line as positive control by WB assay (n = 3). (**F**) The expression of Fpr2 with K562cell line as positive control by WB assay (n = 3). Scale bars: 20 μm.

Immunocytochemistry was used to detect the expression of Fprs by NSCs. Results showed that both Fpr1 and Fpr2 were expressed by NSCs (Fig. 1a,b). RT-PCR assay and WB assay were then used to determine mRNA and protein levels of Fpr1 and Fpr2 in NSCs to corroborate the results obtained with immunocytochemistry. RT-PCR detected a high level expression of Fpr1 and Fpr2 mRNA in NSCs similar to their levels in a positive control cell line K562 (Fig. 1c,d). In addition, WB detected Fpr1 and Fpr2 proteins in NSCs (Fig. 1e,f). Thus, both Fpr1 and Fpr2 are expressed by NSCs.

### Fpr1 and Fpr2 enhance NSC migration both *in vitro* and *in vivo*

**Figure 2:**
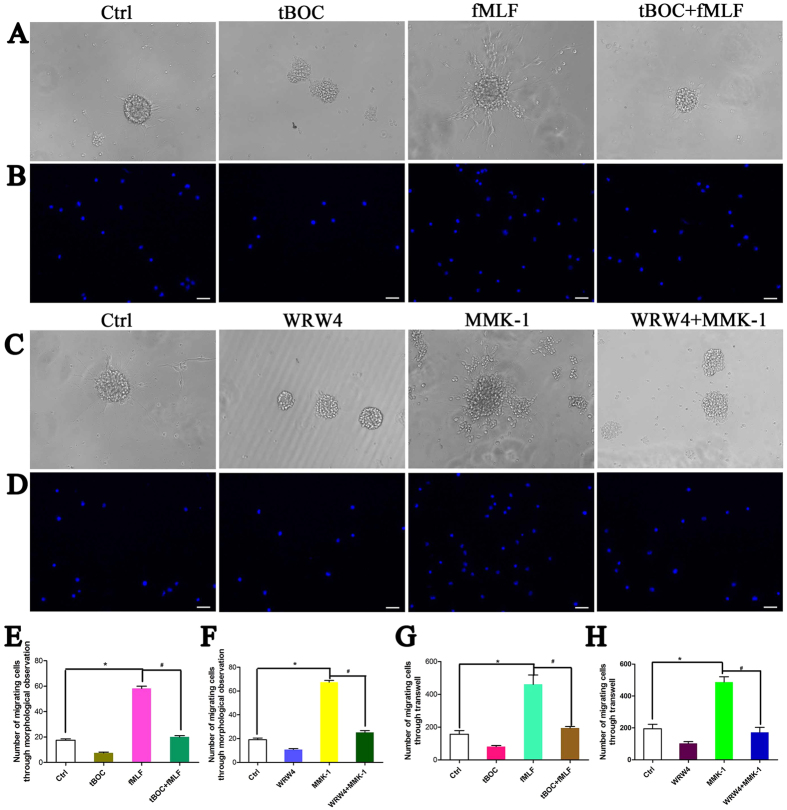
The effects of Fpr1 and Fpr2 on NSCs migration. (**A**) The migration assay with fMLF, tBOC and combination under phase contrast microscope (n = 3). (**B**) The migration assay with fMLF, tBOC and combination with transwell assay (n = 3). (**C**) The migration assay with WRW4, MMK-1 and combination under phase contrast microscope (n = 3). (**D**) The migration assay with WRW4, MMK-1 and combination with transwell assay (n = 3). (**E**) Quantitative assay of migrating cells induced with fMLF, tBOC and combination under phase contrast microscopy. *Significantly increased migrating NSCs in fMLF group as compared with control group (p = 0.0004). ^#^Significantly increased migrating NSCs in fMLF group as compared with tBOC + fMLF group (p = 0.0003). (**F**) Quantitative assay of migrating cells induced with fMLF, tBOC and combination via transwell assay. *Significantly increased migrating NSCs in fMLF group as compared with control group (p = 0.0003). ^#^Significantly increased migrating NSCs in fMLF group as compared with tBOC + fMLF group (p = 0.0007). (**G**) Quantitative assay of migrating cells induced withWRW4, MMK-1 and combination under phase contrast microscopy. *Significantly increased migrated NSCs in MMK-1 group as compared with control group (p = 0.0008). ^#^Significantly increased migrating NSCs in MMK-1 group as compared with WRW4 + MMK-1 group (p = 0.0002).(**H**) Quantitative assay of migrating cells induced withWRW4, MMK-1 and combination via transwell assay. *Significantly increased migrating NSCs in MMK-1 group as compared with control group (p = 0.0003). ^#^Significantly increased migrated NSCs in MMK-1 group as compared withWRW4 + MMK-1 group (p = 0.0004). Scale bar: 20 μm.

**Figure 3:**
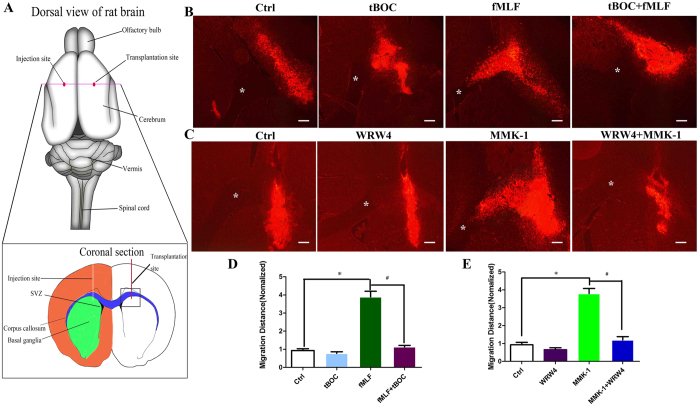
The effect of Fpr1 and Fpr2 on NSCs migration *in vivo*. (**A**) Schematic of cell and reagent injection. fMLF, MMK-1, tBOC and WRW4 were injected into the left subcortical white matter and DiI-labelled NSCs on the opposite point. The areas (boxed) were analyzed on the day 10 after operation. (**B**,**C**) Transplanted NSCs migrate from infusion site, through the corpus callosum toward the midline and the contralateral site of fMLF or MMK-1 infusion. NSC migration is inhibited by tBOC, WRW4. (**D**) Statistical analysis of the distance of NSC migration induced by fMLF. *Significantly increased migration distance in fMLF group as compared with control group (p = 0.0038). ^#^Significantly increased migration distance in fMLF group as compared with tBOC + fMLF group (p = 0.0061). (**E**) Statistical analysis of the distance of NSC migration induced by MMK-1. *Significantly increased migration distance in MMK-1 group as compared with control group (p = 0.0039). ^#^Significantly increased migration distance in MMK-1 group as compared with WRW4 + MMK-1 group (p = 0.0082). Asterisks indicate the corpus callosum. Scale bar: 100 μm.

We then tested the capacity of Fpr1 and Fpr2 to mediate NSC migration by morphological changes, transwell assays *in vitro* and by cell tracking in brain injury model as well as immunohistochemistry *in vivo*. Results showed that Fpr specific agonists induced potent NSC migration *in vitro* and this effect was abrogated by specific Fpr1 or Fpr2 antagonists (Fig. 2). In addition, Fpr1 and Fpr2 synergistically induced NSC migration (Fig. 2e–h). For *in vivo* study, a brain injury model was used to test the migration of transplanted NSCs induced by Fpr agonists. The results clearly showed that prelabelled NSCs migrated away from the infusion site to the midline and the contralateral site of fMLF or MMK-1 infusion site through the corpus callosum. The migration was specifically abrogated by Fpr antagonists (Fig. 3 and Supplementary Figure 1). Immunohistochemistry detection also confirmed the directed migration of endogenous NSC by Fpr agonists *in vivo* (Supplementary Figure 2). Thus, Fprs expressed by NSCs are definitively functional both *in vitro* and *in vivo* and play a pivotal role in the migration of NSCs *in vivo* after CNS injury.

### Fpr1 and Fpr2 promote neuronal differentiation of NSCs

**Figure 4:**
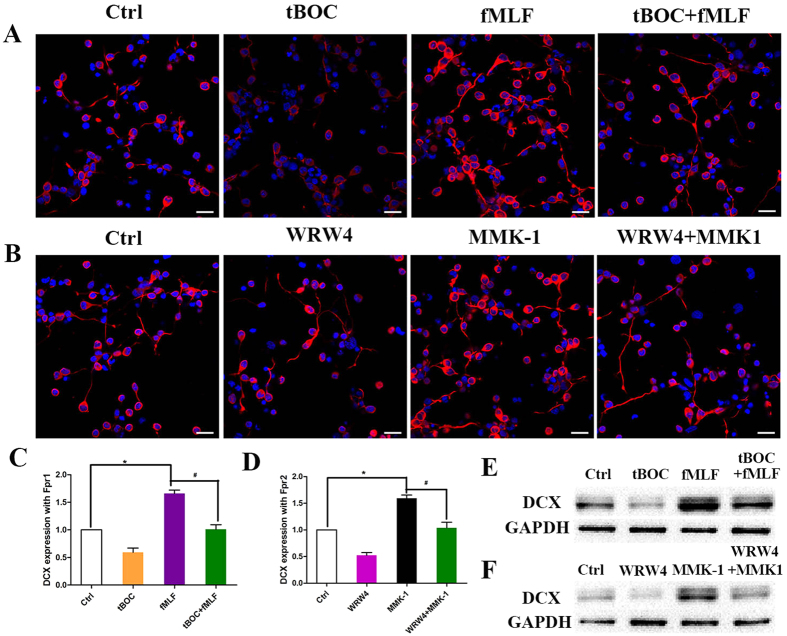
The effect of Fpr1 and Fpr2 on differentiation of NSCs into neurons. (**A**) fMLF, tBOC and combin-ation were used to certify the effect of Fpr1 on NSC differentiation into neurons via immunocytochemistry with DCX (red) and DAPI (blue) (n = 3). (**B**) WRW4, MMK-1 and combination were used to certify the effect of Fpr2 on NSC differentiation into neurons via immunocytochemistry with DCX (red) and DAPI (blue) (n = 3).(**C**) Quantitative assay of neurons induced with fMLF, tBOC and combination. *Significantly increased neurons in fMLF group as compared with control group (p = 0.0006). ^#^Significantly increased neurons in fMLF group as compared with tBOC + fMLF group (p = 0.002). (**D**) Quantitative assay of neurons induced withWRW4, MMK-1 and combination. *Significantly increased neurons in MMK-1 group as compared with control group (p = 0.0007). ^#^Significantly increased neurons in MMK-1 group as compared with WRW4 + MMK-1 group (p = 0.009). (**E**) The expression of DCX with fMLF, tBOC and combination by WB assay. (**F**) The expression of DCX withWRW4, MMK-1 and combination by WB assay. Scale bar: 20 μm.

**Figure 5:**
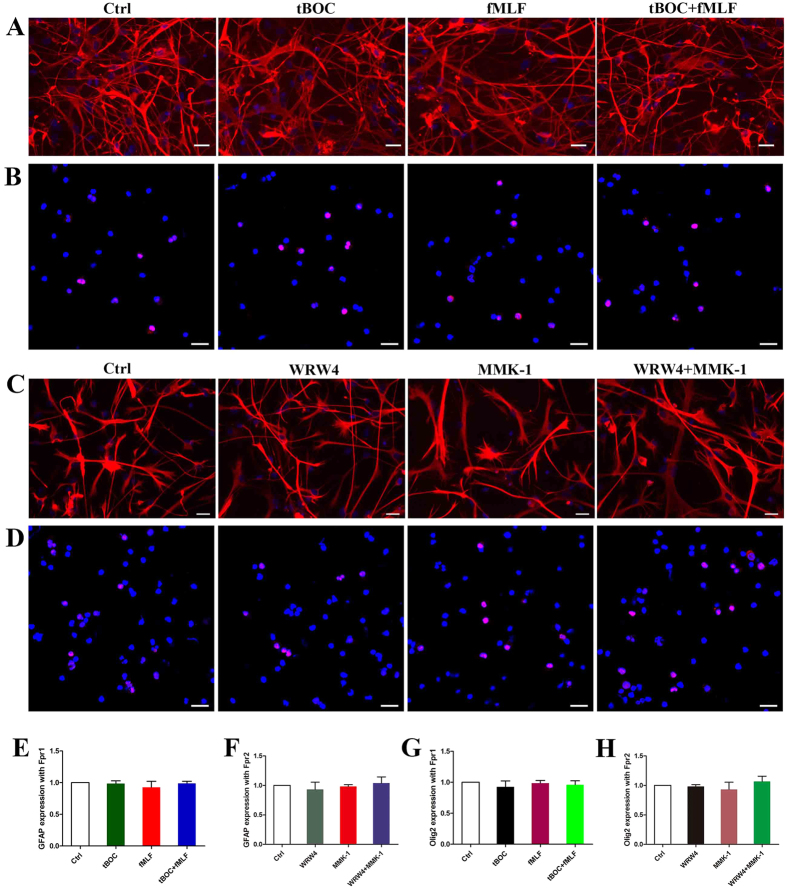
The effect of Fpr1 and Fpr2 on differentiation of NSCs into astrocytes and oligodentrocytes. (**A**) fMLF, tBOC and combination were used to certify the effect of Fpr1 on NSC differentiation into astrocytes via immunocytochemistry with GFAP (red) and DAPI (blue) (n = 3). (**B**) fMLF, tBOC and combination were used to certify the effect of Fpr1 on NSC differentiation into oligodentrocytes via immunocytochemistry with Olig2 (red) and DAPI (blue) (n = 3). (**C**) WRW4, MMK-1 and combination were used to certify the effect of Fpr2 on NSC differentiation into astrocytes via immunocytochemistry with GFAP (red) and DAPI (blue) (n = 3). (**D**) WRW4, MMK-1 and combination were used to certify the effect of Fpr2 on NSC differentiation into oligodentrocytes via immunocytochemistry with Olig2 (red) and DAPI (blue) (n = 3). (**E**) Quantitative assay of astrocytes induced with fMLF, tBOC and combination. (**F**) Quantitative assay of astrocytes induced withWRW4, MMK-1 and combination. (**G**) Quantitative assay of oligodentrocytes induced with fMLF, tBOC and combination. (**H**) Quantitative assay of oligodentrocytes induced with WRW4, MMK-1 and combination. Scale bar: 20 μm.

We next tested whether Fpr1 and Fpr2 promote NSC differentiation of NSCs. Immunocytochemistry indicated that both Fpr1 and Fpr2 skewed NSC differentiation to immature neurons as revealed by significantly increased immature neuron marker DCX 24 hours after stimulation with Fpr1 and Fpr2 agonists (Fig. 4a–d). Mature neuron marker TUJ-1 was markedly increased on day 3 and lasted at least for 7 days after Fpr agonist treatment (Supplementary Figure 3). The effects of Fpr agonists were blocked by antagonists (Figs 3a–d and 4a–d). The expression of DCX was confirmed by WB (Fig. 4e,f). However, Fpr agonists had no effect on NSC differentiation to astrocytes or oligodendrocytes (Fig. 5). We also observed but failed to found any effects of Fpr agonists on the proliferation of NSCs (Supplementary Figure 4).

### Fprs mediate in NSC F-actin polymerization

**Figure 6:**
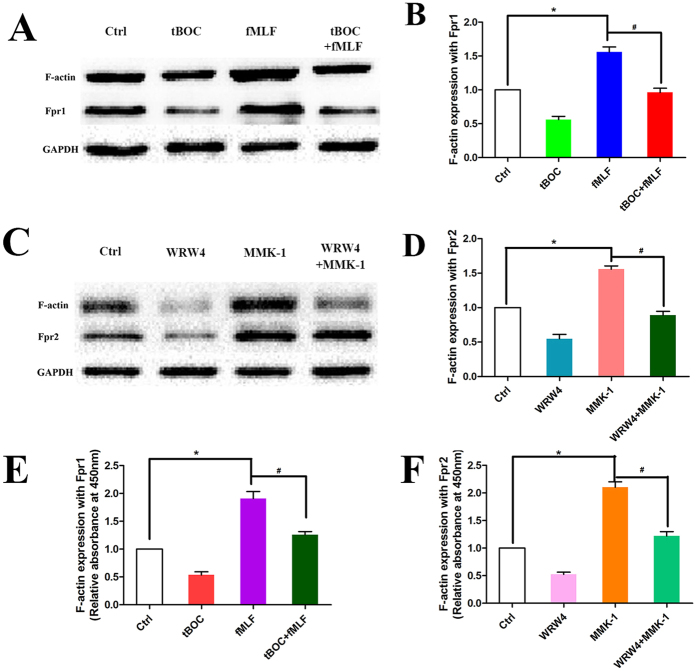
The expression of F-actin in NSCs with Fpr agonist and antagonist. (**A**) WB assay to evaluate the expression of F-actin in NSCs with fMLF, tBOC and combination (n = 3). (**B**) Semi-quantitative assay of F-actin expression in Panel A. *Significantly increased F-actin expression in fMLF group as compared with control group (p = 0.0006). ^#^Significantly increased F-actin expression in fMLF group as compared with tBOC + fMLF group (p = 0.002). (**C**) WB assay to evaluate the expression of F-actin in NSCs with WRW4, MMK-1 and combination (n = 3). (**D**) Semi-quantitative assay of F-actin expression in Panel (**C**). *Significantly increased F-actin expression in MMK-1 group as compared with control group (p = 0.0007). ^#^Significantly increased F-actin expression in MMK-1 group as compared with WRW4 + MMK-1 group (p = 0.0003). (**E**) ELISA assay to evaluate the expression of F-actin in NSCs with fMLF, tBOC and combination (n = 3), *significantly increased F-actin expression in fMLF group as compared with control group (p = 0.002), ^#^significantly increased F-actin expression in fMLF group as compared with tBOC + fMLF group (p = 0.02). (**F**) ELISA assay to evaluate the expression of F-actin in NSCs with WRW4, MMK-1 and combination (n = 3), *significantly increased F-actin expression in MMK-1 group as compared with control group (p = 0.005). ^#^Significantly increased F-actin expression in MMK-1 group as compared with WRW4 + MMK-1 group (p = 0.03).

To examine mechanisms of Fpr1 and Fpr2 mediated NSC migration, we measured the expression of F-actin upon activation of the cells with Fpr agonists. The expression of F-actin was up-regulated in NSCs stimulated with fMLF and MMK-1. The effect of Fpr agonists was inhibited by Fpr antagonists tBOC and WRW4 (Fig. 6). Since F-actin polymerization is required for ligand-induced cell chemotaxis, Fpr-mediated NSC migration is dependent on F-actin.

## Discussion

NSCs bear the capacity for self-renewal and give rise to three main neural cell type(s): neurons, astrocytes and/or oligodendrocytes^1^,^2^, which involve proliferation, differentiation and migration^24^,^25^,^26^. The present study provides the evidence to suggest that NSCs express functional Fprs that promote NSC migration and neuronal differentiation upon stimulation with agonists. This may represent a mechanism for NSCs to home and engraft to the lesions of inflammation and injury in the central nervous system.

Tissue injury, such as cerebral ischemia and/or spinal cord damage, results in inflammation, and leukocyte infiltration^27^. The injured tissue produces inflammatory mediators, such as interleukin-6 (IL-6), tumor necrosis factor alpha (TNF-α), ciliary neurotrophic factor (CNTF), leukemia inhibitory factor (LIF), interferon gamma (IFN-γ), and interleukin-18 (IL-18)^28^,^29^.Among the mediators, monocyte chemoattractant protein-1 (MCP-1, CCL2) and stromal-derived factor-1 (SDF-1,CXCL12) regulate the proliferation, migration and neural differentiation of NSCs^9^,^11^,^30^. Fprs also regulate the function of neutrophils and/or monocytes through the interaction with their ligands^31^,^32^,^33^. Spontaneous migration of NSCs from restricted regions to the lesions is regulated by many molecules. Increasing evidence shows that Fprs are not only expressed by neutrophils and monocytes but are also detected in human brain, spinal cord, anterior horn cells and hypoglossal nucleus neurons^18^. NSCs also interact with some chemokines and cytokines produced by neutrophils and monocytes to enhance their proliferation, migration and final differentiation^6^.

In present study, we showed that both Fpr1 and Fpr2 are expressed on NSCs and both receptors mediate the migration of NSCs *in vitro* and *in vivo*, and promote the differentiation of NSCs to neuron. Consistent with previous findings, both Fpr1 and Fpr2 participate in the normal wound healing process for early neutrophil recruitment and subsequent wound closure^34^. Thus, Fprs may play pivotal roles in NSC migration to lesion sites by interaction with specific ligands in the microenvironment. These results also imply that Fpr ligands could be utilized for the treatment of a variety of clinical conditions, including stroke and spinal cord injury. The present study focuses on the Fpr-mediated migration of primary NSCs. Our results using cell tracking *in vivo* clearly showed that transplanted NSCs migrated in response to Fpr agonists through the corpus callosum. Further studies are needed to identify the subsets of NSCs that utilize Fprs for migration.

Accumulating evidence shows that some signaling pathways coupled to cytokines and chemokines are involved in NSC proliferation, migration and differentiation. The Janus kinase-signal transducer and JAK/STAT alter NSC self-renewal, progenitor cell division and differentiation^28^,^29^. The c-Jun N-terminal kinase (JNK) pathways mediate neuronal differentiation induced by IFN-γ^35^, and also are required for neural differentiation of embryonic carcinoma cells, embryonic stem cells and PC12 cells^36^,^37^,^38^,^39^. Activated microglia secrete insulin-like growth factor-1(IGF-1) under pathological conditions and the subsequent activation of the extracellular signal-regulated kinase (ERK)/mitogen-activated protein kinase (MAPK) pathway increases neurogenesis^40^.

Our study suggests that Fprs may serve as candidates for treatment of brain or spinal cord injury.

## Methods

### Animals and Ethics Statement

All rats used in this study were purchased from experimental animal center of the Third Military Medical University (Chongqing). All animal experiments were approved by Animal Care and Use committee of Third Military Medical University and performed in accordance with the procedures outlined in the “Guide for Care and Use of Laboratory Animals” (Third Military Medical University, Chongqing, China). All efforts had been done to minimize the number of animals and decrease their suffering. E14.5 and 4-week-old male (150–200 g) Sprague-Dawley rats were euthanized with pentobarbital (60 mg/kg intraperitoneal).

### Reagents

All reagents and chemicals were purchased from Sigma-Aldrich (St Louis, MO), unless otherwise specified. Primary antibodies against formylpeptide receptor 1 (Fpr1), formylpeptide receptor 2 (Fpr2), Doublecortin (DCX),beta III Tubulin (TUJ-1), Ki-67 and Nestin were from Abcam (Abcam Trading Company Ltd, Cambridge) and Bioss (Biosynthesis biotechnology Ltd, Beijing), glial fibrillary acidic protein (GFAP) and Olig2 were from Proteintech (Proteintech Group, Inc, Wuhan), glyceraldehyde-3-phosphate dehydrogenase (GAPDH) was from Zsgb (Zsgb-bio, Beijing). F-actin was from Santa Cruz Biotechnology (Santa Cruz, CA). Dulbecco’s Modified Eagle’s Medium (DMEM), DMEM/F-12 medium, fetal bovine serum (FBS) and 0.25% trypsin-EDTA were from Hyclone (Thermo scientific, Logan). B27 and StemProAccutase Cell Dissociation Reagent were from Gibco (Carlsbad, CA). EGF and FGF-2 were from Peprotech (PeproTech, NJ). CM-Dilwas from Cell Tracker (MolecularProbes).

### Primary Neurosphere Culture

We isolated and cultured primary rat NSCs from cortices of fetal E14.5 Sprague–Dawley pups as previously described^41^. Briefly, brains were dissected and incubated in DMEM containing 0.25% trypsin-EDTA and 250U/ml DNase I at 37 °C for 30 min. Then, tissue pieces were washed twice in DMEM with 10% fetal bovine serum to inhibit the digestion of trypsin. The tissue samples were triturated using a fire-polished Pasteur pipette and passed through a 100 μm Nylon cell strainer to harvest dissociated cell suspensions after washed twice with Dulbecco’s Modified Eagle’s Medium. Cell suspensions were and cultured in DMEM/F12 medium supplemented with B27, 20 ng/ml EGF, 20 ng/ml FGF-2 and 1% penicillin-streptomycin at 37 °C under humidified 5% CO_2_ conditions as recommended. For passaging cells, neurospheres were harvested by centrifugation, dissociated in StemProAccutase Cell Dissociation Reagent and grown in the medium described above.

### Immunohistochemistry

For fluorescent immunocytochemistry, neurospheres adhered to poly-ornithine precoated coverslips as previously described. After different treatment, they were incubated in 4% paraformaldehyde in 0.01M phosphate-buffered saline (pH 7.4) for 1 hour at room temperature and blocked with 5% bovine serum album or with 0.3% v/v Triton-X 100 in PBS. Neurospheres were incubated with rabbit polyclonal to Fpr1 (1:200), rabbit polyclonal to Fpr2 (1:200), or Mouse monoclonal to Nestin (1:250) overnight at 4 °C and then incubated in goat anti-rabbit Cy3 or anti-mouse FITC (1:250) for 2 hours at temperature. 4′-6-Diamidino-2-phenylindole (DAPI) was performed to counterstain nuclei for 10 minutes at room temperature. Cover slips were mounted onto glass slides and the image photos were obtained by a fluorescence microscope (BX-34-FLAD1; Olympus) or a confocal microscope (Carl Zeiss LSM510, Germany) and examined by AxioVision4 software.

### Reverse Transcription Polymerase Chain Reaction

Total RNA was extracted from neurospheres with TaKaRaMiniBEST Universal RNA Extraction Kit according to the manufacturer’s instructions (TaKaRa, Japan) and contaminating DNA was depleted with RNase-free DNase (Qiagen, Valencia, CA). Total RNA (2 μg) was reverse transcribed into cDNA with PrimeScript™ II 1st Strand cDNA Synthesis Kit (TaKaRa, Japn) and an aliquot of cDNA mixture (0.2%) was used as polymerase chain reaction (PCR) templates. Primers were as follows: Fpr1(forward, 5′-CAT GAA CAA GTC TGC AGT GAA CCT-3′; reverse, 5′-AGG TTT ATG TCT ATT ACA GTA TAT-3′), Fpr2 (forward, 5′-TCT ACC ATC TCC AGA GTT CTG TGG-3′; reverse, 5′-TTA CAT CTA CCA CAA TGT GAA CTA-3′), GAPDH (forward, 5′-GGC CCC TCT GGA AAG CTG TG-3′; reverse, 5′-CCA GGC GGC ATG GCA GAT C-3′). The annealing temperature for PCR was 56 °C and carried out for 26 cycles. Gels were imaged and digitized with ChemiDoc™ XRS + System (Bio-rad, USA).

### Western blotting

Floating neurospheres and/or neurospheres adhered to poly-ornithine precoated dishes after different treatments were homogenized with lysis buffer containing 0.025M Tris-HCl, pH 8.0, 0.15 M NaCl, 0.001 M EDTA, 1% Nonidet P-40, 10% glycerol; pH 7.4, and a protease inhibitor mixture. Proteins (15 μg per lane) after measured by BCA Protein Assay Kit (Beyotime, Bejing) were separated by 10% SDS-PAGE under reducing conditions and electroblotted to polyvinylidene difluoride membranes (Roche, Basel). The membranes were blocked with 5% (wt/vol) nonfat dry milk in TBST (TBS, 0.1% (vol/vol) Tween 20) for 2 hours at room temperature and subsequently incubated with rabbit polyclonal to Fpr1 (1:2000), rabbit polyclonal to Fpr2 (1:2000), or mouse monoclonal to GAPDH (1:1000) overnight at 4 °C with gentle agitation and peroxidase-conjugated (HRP)-conjugated secondary IgGs (1:5000) for 2 hours at room temperature. All membranes were detected by ChemiDoc™ XRS + imaging system using the Pierce Fast Western Blot Kits (Thermo Scientific, USA). All immunoblots were carried out in duplicates of three independent cell populations and averaged (n = 3).

### ELISA

Quantitative measurement of F-actin and GAPDH were analyzed and determined by the corresponding ELISA kits (Abcam) according to the manufacturer’s instructions. The absorbance was measured at 450 nm.

### Cell Migration Assay *in vitro*

Morphological observation assays were performed with Millipore 96-well cell clusters in accordance with the manufacturer’s instructions. Briefly, about 10–20 μm diameter of 3–5neurosphereswere incubated in 96-well plates in 100 μL DMEM/F12 containing 2%B27 supplemented with 20 ng/ml EGF, 20 ng/ml FGF-2 and 1% penicillin-streptomycin. The agonist and/or antagonist of Fpr1 and Fpr2 were added to each 96-well plates for 12 h at 37 °C in a humidified incubator with 5% CO2.Cell morphology were observed on 12 h and compared with 0 h under the microscope.

Transwell assays were performed with Millipore transwell inserts (8-μm pore filter, 24-well cell clusters) in accordance with the manufacturer’s instructions. The upper chambers were seeded 100 μL (1 × 105/ml) NSCs in DMEM/F-12 medium containing 2% B27 supplemented with 20 ng/ml EGF, 20 ng/ml FGF-2 and 1% penicillin-streptomycin. The lower chambers were filled with 600 μL DMEM/F-12 medium with 6 μM fMLF and 0.5 μM MMK-1 which served as chemoattractants or 0.2 μM tBOC and 0.4 μM WRW4 that served as inhibitors to test Fpr1 and Fpr2 on NSC migration. The NSCs were allowed to migrate from the upper to the lower chambers for 12 h at 37 °C in a humidified incubator with 5% CO2. Non-migratory cells were removed from the top of the membrane with a cotton swab and the cells attached to the lower surface of membrane were fixed in 4% paraformaldehyde at room temperature for 30 min and counterstained with DAPI, and the number was counted under the microscope. A total of 12fields were counted for each transwell filter.

### NSC Migration Assay *in vivo*

A modified cerebral hemorrhage model was used to corroborate the Fpr effects on NSC migration *in vivo*. Firstly, transplanted prelebelled NSCs were observed *in vivo*. Briefly, the animals were anesthetized with 10% Chloral Hydrate. A suspension of 70,000 NSCs cells in 10 μL prelabeled with CM-Dil (Cell Tracker, MolecularProbes, treated according to the instructions of the manufacturer)^7^ was injected into the subcortical white matter on the right (bregma + 0.74 mm, 2.5 mm lateral and 4 mm ventral to skull) used a stereotaxic apparatus. On the same day and the following 10 days, 10 μL containing 6 μM fMLF and 0.5 μM MMK-1 or 0.2 μM tBOC and 0.4 μM WRW4 was injected into the contralateral hemisphere at the same site. Animals were sutured and allowed to recover from surgery on a heating pad, then carefully monitored for the duration of the experiment. On day 10, the brain were sliced and the distance of migration (the distance between the NSC injection site to the furthest point in the corpus callosum and the middle line where the transplanted NSCs reached were analyzed.

In addition, the migration of endogenous NSCs was also detected with immunohistochemistry. 10 μLFpr1 and Fpr2 agonists/antagonists with gradient concentrations were injected into basal ganglia near subventricular zone with fine needle in rat brain. Then, we took out the brain on day 1, 3 and 7 post injection and used immunohistochemistry as previously described to observe NSC migration (Supplementary Figure 2).

### Cell Differentiation Assay

#### Immunocytochemistry

As previously described, cells were incubated with rabbit polyclonal to rabbit polyclonal to Doublecortin (24 h, 1:200), rabbit polyclonal to glial fibrillary acidic protein (GFAP) (24 h, 1:100), rabbit polyclonal to Olig2 (24 h, 1:100,), rabbit polyclonal toTUJ-1 (3d and 7d, 1:200) (n = 3).

#### Western blotting

As previous described, the membranes were subsequently incubated with rabbit polyclonal to Doublecortin (1:2000), or mouse monoclonal to GAPDH (1:1000) (n = 3).

#### Cell Proliferation Assay

The effects of Fpr1, Fpr2 and their agonist and/or antagonist on the NSC proliferation were measured by a CCK-8 assay kit. Briefly, 8 × 103 cells/well were incubated in 96-well plates in 100 μL DMEM/F12 containing B27 supplement without FGF-2 and EGF. The agonist and/or antagonist of Fpr1 and Fpr2 were added every day to each 96-well plates. Cell Counting Kit-8 solution was added after 3 days to each well and incubated for 3 hours. The cell viability in each well was determined by reading the absorbance of the culture medium at a test wavelength of 450 nm and a reference wavelength of 630 nm.

#### CCK-8 assay

The effects of Fpr1, Fpr2 and their agonist and/or antagonist on the NSC proliferation were measured by a CCK-8 assay kit (Dojindo, Japan). Briefly, 8 × 103 cells/well were incubated in 96-well plates in 100 μL DMEM/F12 containing B27 supplement without FGF-2 and EGF. The agonist and/or antagonist of Fpr1 and Fpr2 were added every day to each 96-well plates. Cell Counting Kit-8 solution was added on 1, 3, and 7day to each well and incubated for 3 hours. The cell viability in each well was determined by reading the absorbance of the culture medium at a test wavelength of 450 nm and a reference wavelength of 630 nm (Thermo Scientific, USA).

Ki-67 assays were performed with immunocyto chemistry as previously described which incubated with rabbit polyclonal to Ki-67 (1:200) or Mouse monoclonal to Nestin (1:250).

#### Statistical analysis

All data were presented as mean ± SEM and statistical analyses were performed with GraphPad Prism v5.0 (GraphPad Software, Inc. USA).Statistical differences among testing and control groups were analyzed by Student’s t-test and one-way analysis of variance (ANOVA) followed by Bonferroni’s multiple comparison post-test. A P value < 0.05 was considered.

## Additional Information

**How to cite this article**: Wang, G. *et al.* Formylpeptide Receptors Promote the Migration and Differentiation of Rat Neural Stem Cells. *Sci. Rep.* **6**, 25946; doi: 10.1038/srep25946 (2016).

## Supplementary Material

Supplementary Information

## References

[b1] Gage F. H. Mammalian neural stem cells. Science 287, 1433–1438 (2000). 10688783 10.1126/science.287.5457.1433

[b2] Ming G. L. & Song H. Adult neurogenesis in the mammalian brain: significant answers and significant questions. Neuron 70, 687–702 (2011). 21609825 10.1016/j.neuron.2011.05.001 PMC3106107

[b3] Arvidsson A., Collin T., Kirik D., Kokaia Z. & Lindvall O. Neuronal replacement from endogenous precursors in the adult brain after stroke. NatMed 8, 963–970 (2002). 10.1038/nm747 12161747

[b4] Astrup J., Siesjo B. K. & Symon L. Thresholds in cerebral ischemia-the ischemic penumbra. Stroke 12, 723–725 (1981). 6272455 10.1161/01.str.12.6.723

[b5] Briona L. K. & Dorsky R. I. Radial glial progenitors repair the zebrafish spinal cord following transection. Exp Neurol 256, 81–92 (2014). 24721238 10.1016/j.expneurol.2014.03.017 PMC4038170

[b6] Gonzalez-Perez O., Jauregui-Huerta F. & Galvez-Contreras A. Y. Immune system modulates the function of adult neural stem cells. Curr Immunol Rev 6, 167–173 (2010). 21037937 10.2174/157339510791823772 PMC2964894

[b7] Imitola J. *et al.* Directed migration of neural stem cells to sites of CNS injury by the stromal cell-derived factor 1alpha/CXC chemokine receptor 4 pathway. Proc Natl Acad Sci USA 101, 18117–18122 (2004). 15608062 10.1073/pnas.0408258102 PMC536055

[b8] Thored P. *et al.* Persistent production of neurons from adult brain stem cells during recovery after stroke. Stem cells 24, 739–747 (2006). 16210404 10.1634/stemcells.2005-0281

[b9] Widera D. *et al.* MCP-1 induces migration of adult neural stem cells. Eur J Cell Biol 83, 381–387 (2004). 15506562 10.1078/0171-9335-00403

[b10] Peng H. *et al.* Stromal cell-derived factor 1-mediated CXCR4 signaling in rat and human cortical neural progenitor cells. J Neurosci Res 76, 35–50 (2004). 15048928 10.1002/jnr.20045

[b11] Molyneaux K. A. *et al.* The chemokine SDF1/CXCL12 and its receptor CXCR4 regulate mouse germ cell migration and survival. Development 130, 4279–4286 (2003). 12900445 10.1242/dev.00640

[b12] Gao J. L., Chen H., Filie J. D., Kozak C. A. & Murphy P. M. Differential expansion of the N-formylpeptide receptor gene cluster in human and mouse. Genomics 51, 270–276 (1998). 9722950 10.1006/geno.1998.5376

[b13] Bao L., Gerard N. P., Eddy R. L. Jr., Shows T. B. & Gerard C. Mapping of genes for the human C5a receptor (C5AR), human FMLP receptor (FPR), and two FMLP receptor homologue orphan receptors (FPRH1, FPRH2) to chromosome 19. Genomics 13, 437–440 (1992). 1612600 10.1016/0888-7543(92)90265-t

[b14] Murphy P. M. *et al.* A structural homologue of the N-formyl peptide receptor. Characterization and chromosome mapping of a peptide chemoattractant receptor family. J Biol Chem 267, 7637–7643 (1992). 1373134

[b15] Ye R. D., Cavanagh S. L., Quehenberger O., Prossnitz E. R. & Cochrane C. G. Isolation of a cDNA that encodes a novel granulocyte N-formyl peptide receptor. Biochem Biophys Res Commun 184, 582–589 (1992). 1374236 10.1016/0006-291x(92)90629-y

[b16] Le Y. *et al.* Expression of functional formyl peptide receptors by human astrocytoma cell lines. J Neuroimmunol 111, 102–108 (2000). 11063827 10.1016/s0165-5728(00)00373-8

[b17] Sozzani S. *et al.* Migration of dendritic cells in response to formyl peptides, C5a, and a distinct set of chemokines. J Immunol 155, 3292–3295 (1995). 7561021

[b18] Becker E. L. *et al.* Broad immunocytochemical localization of the formylpeptide receptor in human organs, tissues, and cells. Cell Tissue Res 292, 129–135 (1998). 9506920 10.1007/s004410051042

[b19] Merino J. J., Bellver-Landete V., Oset-Gasque M. J. & Cubelos B. Review: CXCR4/CXCR7 molecular involvement in neuronal and neural progenitor migration: focus in CNS repair. J Cell Physiol 230, 27–42 (2014). 24913264 10.1002/jcp.24695

[b20] Perez H. D., Kelly E., Elfman F., Armitage G. & Winkler J. Defective polymorphonuclear leukocyte formyl peptide receptor(s) in juvenile periodontitis. J Clin Invest 87, 971–976 (1991). 1999504 10.1172/JCI115105 PMC329889

[b21] Stenfeldt A. L. *et al.* Cyclosporin H, Boc-MLF and Boc-FLFLF are antagonists that preferentially inhibit activity triggered through the formyl peptide receptor. Inflammation 30, 224–229 (2007). 17687636 10.1007/s10753-007-9040-4

[b22] Hu J. Y. *et al.* Synthetic peptide MMK-1 is a highly specific chemotactic agonist for leukocyte FPRL1. J Leukoc Biol 70, 155–161 (2001). 11435499

[b23] Fu H. *et al.* The two neutrophil members of the formylpeptide receptor family activate the NADPH-oxidase through signals that differ in sensitivity to a gelsolin derived phosphoinositide-binding peptide. BMC Cell Biol 5, 50 (2004). 15625007 10.1186/1471-2121-5-50 PMC545074

[b24] Paton J. A. & Nottebohm F. N. Neurons generated in the adult brain are recruited into functional circuits. Science 225, 1046–1048 (1984). 6474166 10.1126/science.6474166

[b25] Luo J. *et al.* Physical exercise regulates neural stem cells proliferation and migration via SDF-1alpha/CXCR4 pathway in rats after ischemic stroke. Neurosci Lett 578, 203–8 (2014). 25010020 10.1016/j.neulet.2014.06.059

[b26] Obernier K., Tong C. K. & Alvarez-Buylla A. Restricted nature of adult neural stem cells: re-evaluation of their potential for brain repair. Front Neurosci 8, 162 (2014). 24987325 10.3389/fnins.2014.00162 PMC4060730

[b27] Clark R. K. *et al.* Reperfusion following focal stroke hastens inflammation and resolution of ischemic injured tissue. Brain Res Bull 35, 387–392 (1994). 7850491 10.1016/0361-9230(94)90119-8

[b28] Miller R. J. *et al.* Chemokine action in the nervous system. J Neurosci 28, 11792–11795 (2008). 19005041 10.1523/JNEUROSCI.3588-08.2008 PMC2746239

[b29] Bauer S. Cytokine control of adult neural stem cells. Ann N Y Acad Sci 1153, 48–56 (2009). 19236327 10.1111/j.1749-6632.2009.03986.x

[b30] Schwamborn J. *et al.* Microarray analysis of tumor necrosis factor alpha induced gene expression in U373 human glioblastoma cells. BMC genomics 4, 46 (2003). 14641910 10.1186/1471-2164-4-46 PMC317285

[b31] Pupjalis D., Goetsch J., Kottas D. J., Gerke V. & Rescher U. Annexin A1 released from apoptotic cells acts through formyl peptide receptors to dampen inflammatory monocyte activation via JAK/STAT/SOCS signalling. EMBO Mol Med 3, 102–114 (2011). 21254404 10.1002/emmm.201000113 PMC3377061

[b32] Girol A. P. *et al.* Anti-inflammatory mechanisms of the annexin A1 protein and its mimetic peptide Ac2-26 in models of ocular inflammation *in vivo* and *in vitro*. J Immunol 190, 5689–5701 (2013). 23645879 10.4049/jimmunol.1202030

[b33] Bizzarro V. *et al.* Annexin A1 N-terminal derived peptide Ac2-26 stimulates fibroblast migration in high glucose conditions. PloS one 7, e45639 (2012). 23029153 10.1371/journal.pone.0045639 PMC3448638

[b34] Liu M. *et al.* Formylpeptide receptors mediate rapid neutrophil mobilization to accelerate wound healing. PloS one 9, e90613 (2014). 24603667 10.1371/journal.pone.0090613 PMC3946181

[b35] Kim S. J. *et al.* Interferon-gamma promotes differentiation of neural progenitor cells via the JNK pathway. Neurochem Res 32, 1399–1406 (2007). 17415631 10.1007/s11064-007-9323-z

[b36] Zentrich E., Han S. Y., Pessoa-Brandao L., Butterfield L. & Heasley L. E. Collaboration of JNKs and ERKs in nerve growth factor regulation of the neurofilament light chain promoter in PC12 cells. J Biol Chem 277, 4110–4118 (2002). 11733514 10.1074/jbc.M107824200

[b37] Akiyama S. *et al.* Activation mechanism of c-Jun amino-terminal kinase in the course of neural differentiation of P19 embryonic carcinoma cells. J Biol Chem 279, 36616–36620 (2004). 15218018 10.1074/jbc.M406610200

[b38] Wang H. *et al.* Activation of c-Jun amino-terminal kinase is required for retinoic acid-induced neural differentiation of P19 embryonal carcinoma cells. FEBS Lett 503, 91–96 (2001). 11513861 10.1016/s0014-5793(01)02699-0

[b39] Amura C. R., Marek L., Winn R. A. & Heasley L. E. Inhibited neurogenesis in JNK1-deficient embryonic stem cells. Mol Cell Biol 25, 10791–10802 (2005). 16314504 10.1128/MCB.25.24.10791-10802.2005 PMC1316944

[b40] Choi Y. S., Cho H. Y., Hoyt K. R., Naegele J. R. & Obrietan K. IGF-1 receptor-mediated ERK/MAPK signaling couples status epilepticus to progenitor cell proliferation in the subgranular layer of the dentate gyrus. Glia 56, 791–800 (2008). 18338791 10.1002/glia.20653 PMC4152854

[b41] Liu Y. *et al.* Directed differentiation of forebrain GABA interneurons from human pluripotent stem cells. Nat Protoc 8, 1670–1679 (2013). 23928500 10.1038/nprot.2013.106 PMC4121169

