# Supplementary material for: Formylpeptide Receptors Promote the Migration and Differentiation of Rat Neural Stem Cells
Source: Sci Rep. 2016 May 13;6:25946. doi: 10.1038/srep25946 (PMC4865803; doi:10.1038/srep25946)
Supplement: Supplementary Information [file srep25946-s1.pdf]

Formylpeptide Receptors Promote the Migration and Differentiation of Rat Neural Stem Cells

Guan Wang[1], Liang Zhang[1], Xingxing Chen[1], Xin Xue[1], Qiaonan Guo[2], Mingyong Liu[1*] & Jianhua Zhao[1*]

[1]Department of Spine Surgery, Daping Hospital, Third Military Medical University, Chongqing 400042, China, [2]Department of Pathology, Xinqiao Hospital, Third Military Medical University, Chongqing 400037, China.

Supplementary Figures

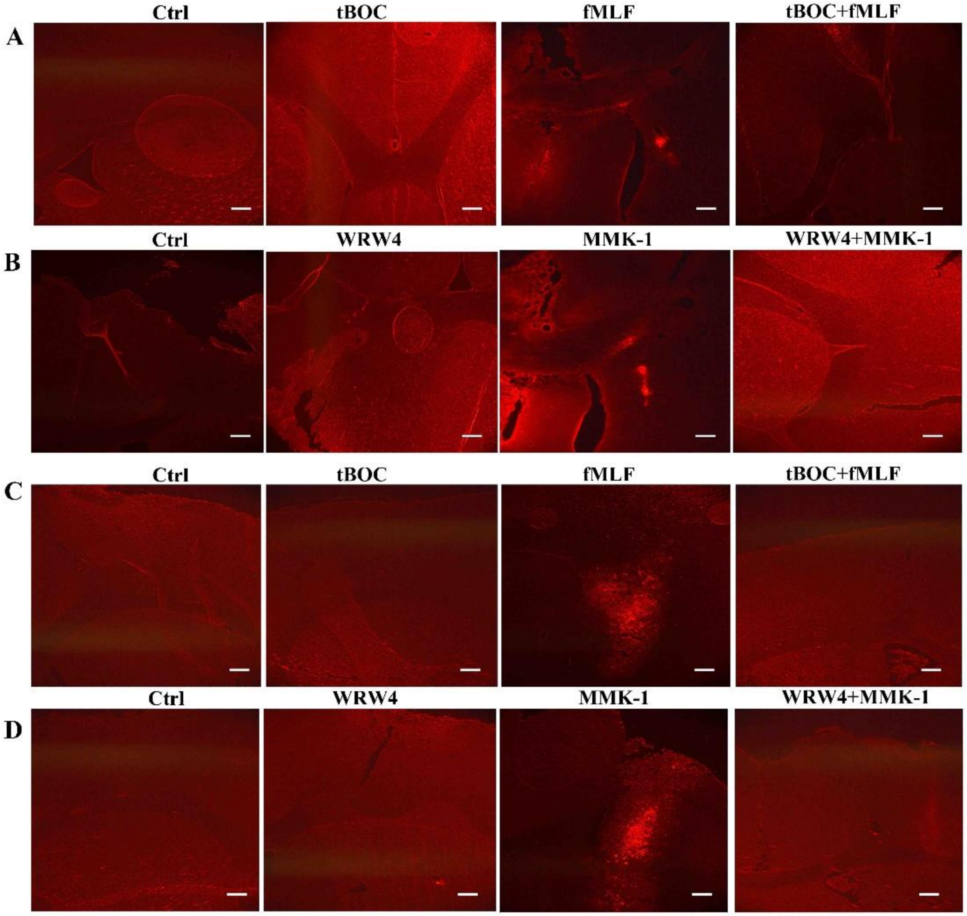

Supplementary Figure 1 | The effect of Fpr1 and Fpr2 on NSCs migration in vivo. Photomicrographs of the corpus callosum (A, B) and infusion sites (C, D). Lack of NSCs migration toward vehicle, tBOC , WRW4, fMLF+tBOC and MMK-1+WRW4 . NSCs migration away from transplantation site(As shown in Fig.3), through the corpus callosum(A and B) toward the midline and the contralateral site(C and D) of fMLF or MMK-1 infusion. Scale bar: 100μm

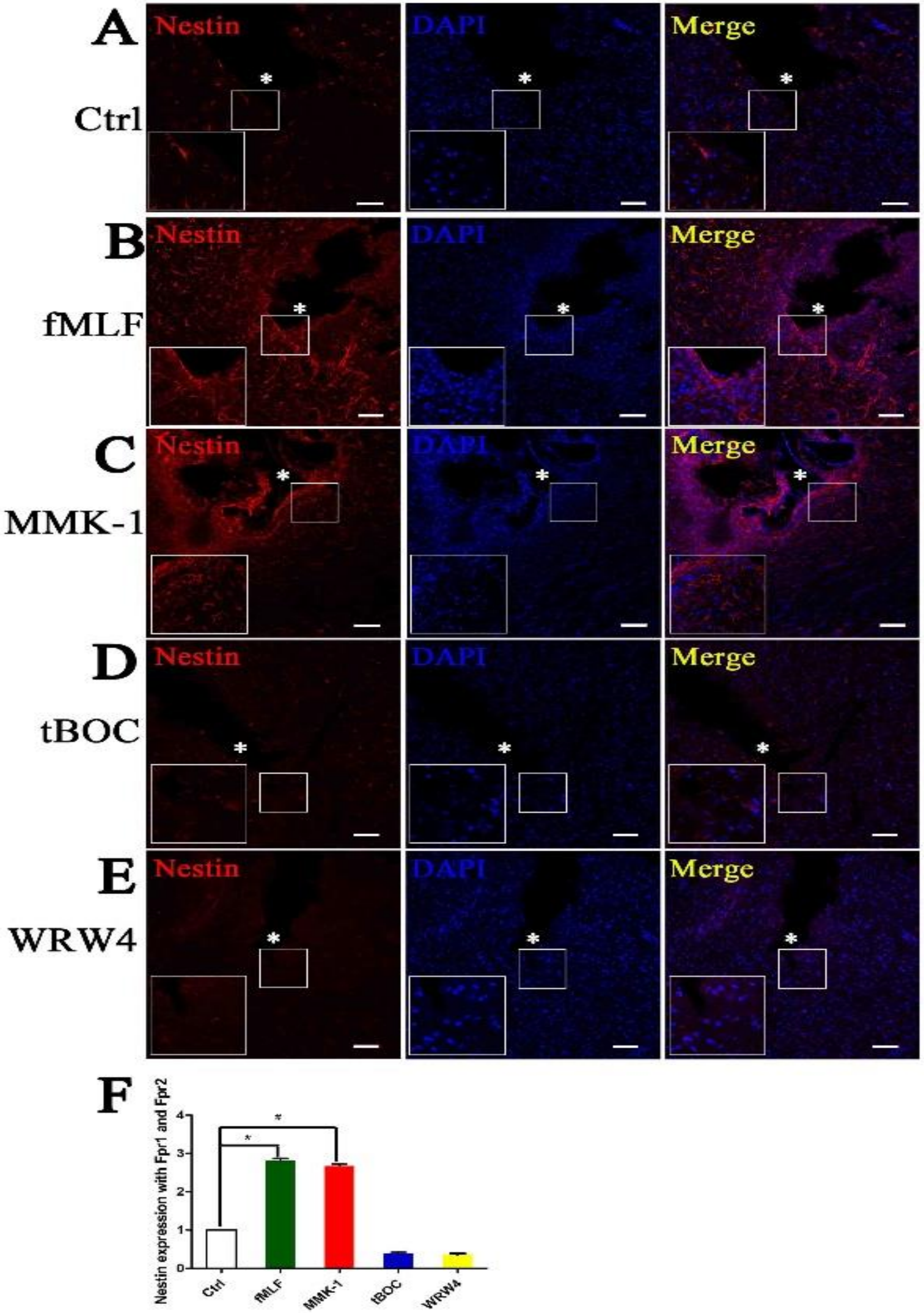

Supplementary Figure 2 | The migration assay with ctrl, fMLF, MMK-1, tBOC and WRW4 on day 7 (n=3). (A - E)Solvent for Fpr agonists and antagonists (DD water) was used as Ctrl. Brain tissue of injection site was sectioned and stained with Nestin (red) and DAPI (blue)to show migrating NSCs, *shows injection site. (B, D) fMLF and tBOC used to certify the effect of Fpr1 on NSC migration. (C, E)MMK-1 and WRW4was used to certify the effect of Fpr2 on NSC migration. (F) Quantitative assay in Nestin expression induced with fMLF, MMK-1, tBOC and WRW4, *significantly increased Nestin expression in fMLF group as compared with ctrl group (p=0.0001), #significantly increased Nestin expression in MMK-1 group as compared with ctrl group (p=0.0004). Scale bar: 100μm.

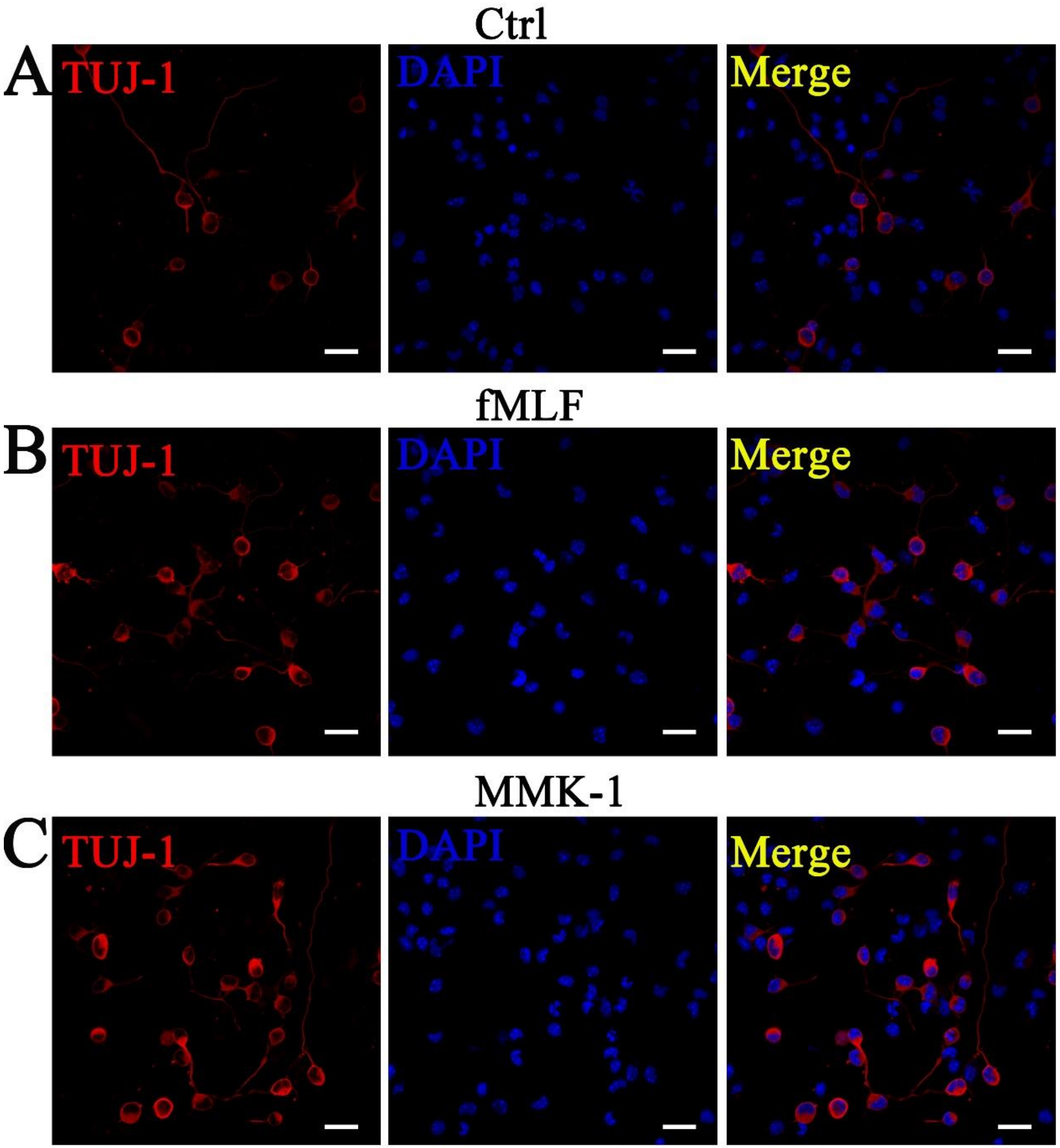

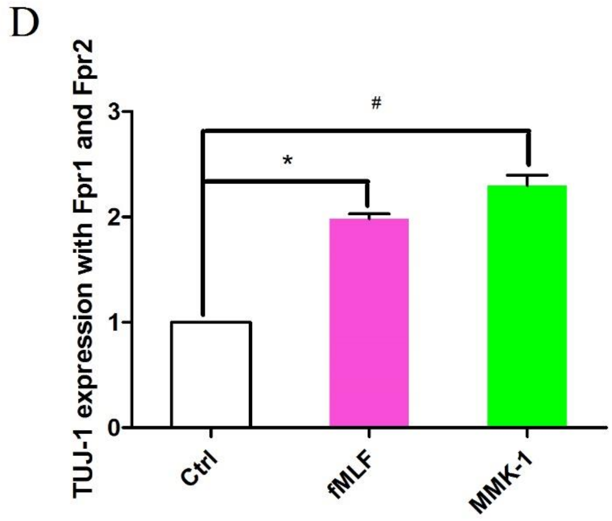

Supplementary Figure 3 | The effect of Fpr1 and Fpr2 on the expression of TUJ-1. (A)TUJ-1 expression in NSCs without Fpr agonists. (B) TUJ-1 expression in NSCs stimulated with Fpr1 agonist fMLF. (C) TUJ-1 expression in NSCs stimulated with Fpr2 agonist MMK-1.(D) Semi-quantitative data of panels A-C. Scale bar: 20μm.

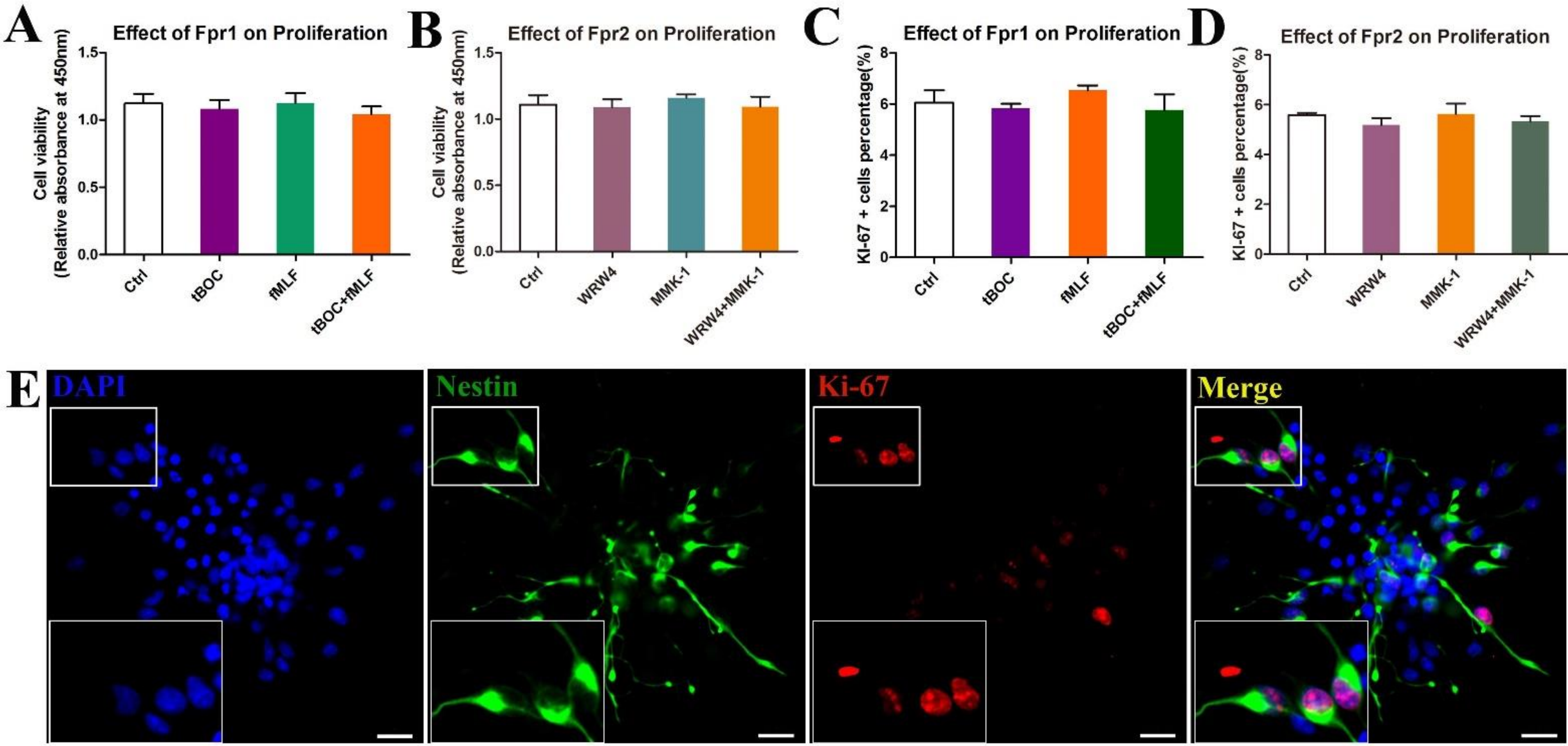

Supplementary Figure 4 | The effect of Fpr1 and Fpr2 on the proliferation of NSCs. (A) fMLF, tBOC and combination were used to certify the effect of Fpr1 on NSC proliferation via CCK-8 assay (n=6). (B) WRW4, MMK-1 and combination were used to certify the effect of Fpr2 on NSC proliferation via CCK-8 assay (n=6). (C) fMLF, tBOC and combination were used to certify the effect of Fpr1 on NSC proliferation via Ki-67 immunocytochemistry (n=3). (D) WRW4, MMK-1 and combination were used to certify the effect of Fpr2 on NSC proliferation via Ki-67 immunocytochemistry (n=3). (E) The effect of Fpr1on NSC proliferation using Ki-67 immunocytochemistry with Nestin (green), Ki-67 (red) and DAPI (blue). Scale bar: 20μm
